# Experimental Investigation on 3D Graphene-CNT Hybrid Foams with Different Interactions

**DOI:** 10.3390/nano8090694

**Published:** 2018-09-06

**Authors:** Hye-soo Kim, Stephanie K. Lee, Mei Wang, Junmo Kang, Yan Sun, Jae Wook Jung, Kyunghoon Kim, Sung-Min Kim, Jae-Do Nam, Jonghwan Suhr

**Affiliations:** 1 Department of Polymer Science and Engineering, Sungkyunkwan University, Suwon 16419, Korea; haesooo5516@gmail.com (H.-s.K.); jdnam@skku.edu (J.-D.N.); 2 Department of Energy Science, Sungkyunkwan University, Suwon 16419, Korea; stephklee@skku.edu (S.K.L.); sunyan@skku.edu (Y.S.); 3 Institute of Laser Spectroscopy, Shanxi University, Taiyuan 030006, China; wangmei@sxu.edu.cn; 4 Department of Materials Science and Engineering, Northwestern University, Evanston, IL 60208, USA; junmo9000@northwestern.edu; 5 B.H.K., Sasang-gu, Busan 46948, Korea; omaeya@nate.com; 6 School of Mechanical Engineering, Sungkyunkwan University, Suwon 16419, Korea; kenkim@skku.edu (K.K.); smkim@skku.edu (S.-M.K.)

**Keywords:** graphene oxide, carbon nanotube, hybrid foam, surfactants, charge effect

## Abstract

Due to the exceptional properties of graphene, numerous possibilities for real applications in various fields have been provided. However, it is a challenge to fabricate bulk graphene materials with properties arising from the nature of individual graphene sheets, and which assemble into monolithic three-dimensional structures. If 3D structured graphene foam were made instead of 2D structured graphene, it is expected that it would be a facile fabrication, with relatively low cost with the possibility of scale-up, and would maintain the intrinsic properties of graphene. To solve the weaknesses of 2D structured graphene, this study aimed to fabricate a 3D graphene-carbon nanotubes (CNT) hybrid foam. In this study, CNT was used to reinforce the graphene foams. In addition, two different surfactants, known as sodium dodecylbenzene sulphonate (SDBS) and cetyltrimethylammonium bromide (CTAB), were applied to help CNT dispersion. The π–π interaction was induced by SDBS/CNT, while ionic interaction was derived from CTAB/CNT. To confirm the charge effect with different surfactants, SEM, Zeta-potential, FT-IR, Raman spectroscopy, and compression tests were performed. When using a cationic surfactant, CTAB, compressive modulus, and strength increased due to the formation of relatively strong ionic bonding.

## 1. Introduction

Graphene has come into the spotlight owing to its extraordinary properties [1,2,3,4,5]. Graphene plays a significant role in a variety of fields, such as electronics, biomedical, energy storage devices, sensors, and other fundamental research fields [6,7]. Although graphene is the driving force behind various fields, it has several drawbacks, which are non-effective mass production, high cost, difficulty in controlling high-quality materials, defects, dispersion problems, and interphase of matrix-filler when becoming composite materials, and so on [8].

Recent studies have revealed that 3D graphene architectures have arisen as a keyword to resolve the aforementioned negative points of fabrication of graphene [9]. Otherwise, it would not be possible to translate the outstanding properties of 2D graphene at an industrial level. To improve some drawbacks, it is helpful to use chemical exfoliation [10,11]. However, challenges still exist with the 3D graphene architectures. In some researches on 3D graphene, the template-directed process was used to fabricate 3D graphene architectures. They succeeded in fabricating 3D graphene architectures which grew on a nickel foam, but they needed additional metal foams and struggled to fabricate a film form [12,13]. Another research reported the graphene oxide (GO) coated polyurethane foam [14]. Furthermore, a GO-based polymeric foam was demonstrated to exhibit a large compressive strain where the maximum achieved stress in the range of 0.4 MPa under compression [15].

Herein, a strong graphene foam produced by introducing carbon nanotubes (CNTs) as reinforcement is suggested. CNTs have been attracting significant interest as reinforcement in composites as well as in graphene due to their various unique properties [16,17,18,19,20]. To confirm the “charge effect” depending on ions, a strategy for two different surfactants was designed for dispersion. Sodium dodecylbenzene sulphonate (SDBS) is an anionic surfactant, while cetyltrimethylammonium bromide (CTAB) is a cationic surfactant in aqueous solution and at the air-water interface. As these two different surfactants have different charges, these can lead to different interaction with the GO sheet.

Hence, to achieve the facile fabrication, a modified Hummers’ method was applied to synthesize of GO. Ethylenediamine (EDA) acts as a reducing agent to functionalize and connect the GO sheets. These foams are fabricated by subjecting a wet-gel precursor to freeze-drying to remove the ice crystals without collapsing the cell structure. SEM was measured to identify structural and morphological changes with low and high magnification. In addition, Raman, Zeta-potential, and FT-IR were conducted to identify a good connection with each different surfactant on CNTs. Finally, compression tests under compressive stress were performed, and the modulus and strength were increased by 157% and 54.3%, respectively.

## 2. Materials and Methods

### 2.1. Materials

Graphite, sulfuric acid (H_2_SO_4_, SAMCHUN, Seoul, Korea, 95%), potassium permanganate (KMnO_4_, Sigma Aldrich, St. Louis, MI, USA), sodium nitrite (NaNO_2_, Sigma Aldrich, >99%), hydrogen chloride (HCl, SAMCHUN), hydrogen peroxide (H_2_O_2_, SAMCHUN, 30%), single-walled carbon nanotubes (SWNT, HiPco, Carbon Nanotechnologies Inc. (CNI), Houston, TX, USA), SDBS (Sigma Aldrich), and CTAB (Sigma Aldrich) were used to synthesize and fabricate the graphene oxide and 3D graphene-CNT hybrid foams.

### 2.2. Synthesis of Graphene Oxide

Graphite oxide was prepared via a modified Hummers’ method [21,22]. 0.6 g of graphite powder was added into 46 mL of concentrated H_2_SO_4_ at 0 °C in an ice bath. After vigorous stirring, the mixture of graphite powder and H_2_SO_4_ was removed from the ice bath, and then, 1.0 g of NaNO_2_ and 3.0 g of KMnO_4_ were slowly added to the mixture. The temperature of the mixture was controlled under 10 °C while adding chemicals to avoid the reaction inside of the mixture. Then, 80 mL of H_2_O was added to the mixture slowly. After 30 min, 800 mL of H_2_O, 6 mL of 30% H_2_O_2_ aqueous solution and 5 mL of HCl were added into the deep brown mixture with vigorous stirring. After cooling the mixture to room temperature, the resulting suspension was centrifuged to it wash out, and this was repeated until it became neutral (pH ~ 7). Finally, the resulting sediment was dried in the freeze-dryer.

### 2.3. Preparation of CNT Dispersion

**Figure 1:**
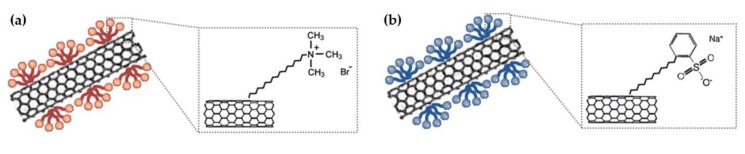
Schematic of chemical interaction for the graphene-carbon nanotubes (CNT) with different surfactants: (**a**) cetyltrimethylammonium bromide (CTAB); (**b**) sodium dodecylbenzene sulphonate (SDBS).

Before the preparation of CNT dispersion, 1 g of each surfactant, such as SDBS and CTAB, was ultra-sonicated with 100 mL of H_2_O for 5 min. Then, 0.1 g of SWNT was added with this solution under ultrasonic agitation for 3 h at room temperature with the aim of making a good dispersion. Finally, the dispersed SWNT solution was centrifuged for 30 min to collect the supernatants. The chemical interaction between CNT and surfactants (SDBS and CTAB) is illustrated in Figure 1.

### 2.4. Fabrication of Graphene-CNT Hybrid Foams

**Figure 2:**
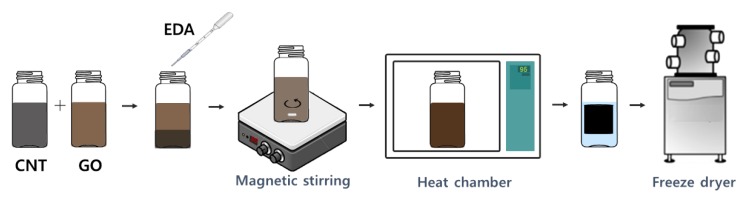
Schematic of an experimental process for the graphene-CNT hybrid foam fabrication.

The synthesis process of the reduced graphene foam is illustrated in Figure 2 [9]. Ethylenediamine (24 μL) was added into the mixture of GO and CNT dispersion. The mixture was sealed in a glass vial and stirred with a magnetic stirrer. Moreover, it was heated for 6 h at 95 °C for the synthesis of the functionalized graphene-CNT hybrid hydrogel. After freeze-drying for 48 h, the solvent in the hydrogel was completely removed.

### 2.5. Characterization

The cell structures of the graphene foam and graphene-CNT hybrid foam were measured by scanning electron microscopy (SEM, JSM6700F, JEOL, Tokyo, Japan). To investigate the interaction for the dispersion of GO and GO-CNT, and the chemical bonding between the CNT with each surfactant and GO, the Fourier transform infrared spectroscopy (FT-IR, IFS-66/S, Bruker, Billerica, MA, USA) within a range of 500–4000 cm^−1^ and Raman spectroscopy (Senterra Raman, Bruker, Billerica, MA, USA) within a range of 400–4000 cm^−1^ were used, respectively. In addition, the surface charges (zeta potential) of the samples were measured by Malvern instrument (Malvern, UK). Finally, compression tests were conducted to evaluate mechanical properties under compression (DMA Q800, TA Instruments, New Castle, DE, USA). The specimens were cylindrical in shape and had dimensions of 5 mm of diameter and 2.5 mm in height. Tests were conducted along with ASTM 1621-10, while strain rate is 0.3 mm/min.

## 3. Results and Discussion

**Figure 3:**
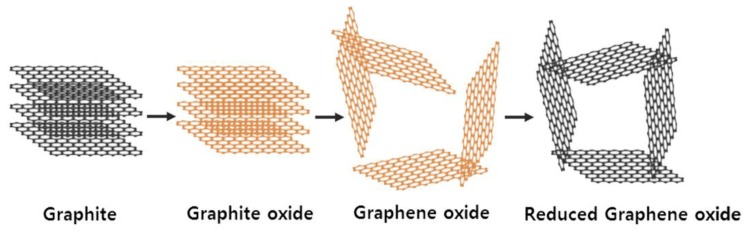
Illustration of self-assembly mechanism for 3D graphene architecture during a chemical reduction of graphene oxide (GO).

3D graphene architecture, called reduced graphene oxide, was prepared via self-assembly (Figure 3). GO has hydrophilicity due to the formation of functional groups, such as hydroxyl, carboxyl, and epoxy groups, during oxidation of graphite. Thus, GO can be dispersed well into the water, and the reduction of GO by EDA in an aqueous suspension results in the formation of reduced GO with hydrophobicity [23,24]. During reduction, the hydrophobicity of GO increased, and self-assembly occurred through π–π stacking interactions creating a reduced GO hydrogel. As a result, it formed a compact 3D graphene architecture due to the steric hindrance effect of the reduced GO sheets.

**Figure 4:**
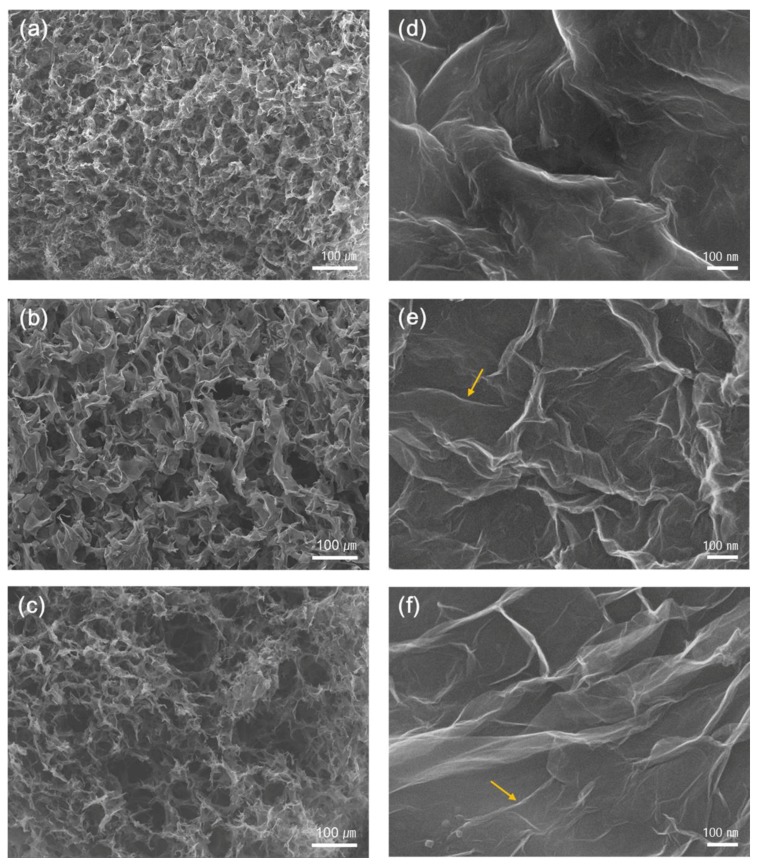
Low magnification SEM images of the cellular structure of GO (**a**), graphene oxide-sodium dodecylbenzene sulphonate/single-walled carbon nanotubes (GO-SDBS/SWNT) (**b**) and graphene oxide-cetyltrimethylammonium bromide/single-walled carbon nanotubes (GO-CTAB/SWNT) (**c**), respectively; High magnification SEM images of GO (**d**), GO-SDBS/SWNT (**e**), and GO-CTAB/SWNT (**f**), respectively.

As shown in Figure 4, SEM images demonstrate the morphologies of hybrid foams incorporating GO (Figure 4a,d), graphene oxide-sodium dodecylbenzene sulphonate/single-walled carbon nanotubes (GO-SDBS/SWNT) (Figure 4b,e) and graphene oxide-cetyltrimethylammonium bromide/single-walled carbon nanotubes (GO-CTAB/SWNT) (Figure 4c,f), respectively. From the SEM images with a low magnification as shown in Figure 4a–c, the structure of interconnected networks ranging from ten to hundreds of micrometers is affirmed. While GO foam has pores less than a hundred micrometer, the pore size of GO-SWNT hybrid foams, such as GO-SDBS/SWNT and GO-CTAB/SWNT, is larger than that of GO foam. This resulted from well-dispersed SWNTs covered with a surfactant [25].

Figure 4d–f shows SEM images with high magnification. These hybrid aerogels show a porous 3D network of assembled sheet-like structures produced during the freezing process. Upon freezing, these individual GO sheets are assembled with the formation of ice crystals. Moreover, the in-plane size of these assembled sheets can lead to an elastic stiffness, up to tens of micrometers. Compared with GO foam, randomly oriented SWNTs can be found for two kinds of GO-SWNT hybrid foams as shown in Figure 4e,f.

**Figure 5:**
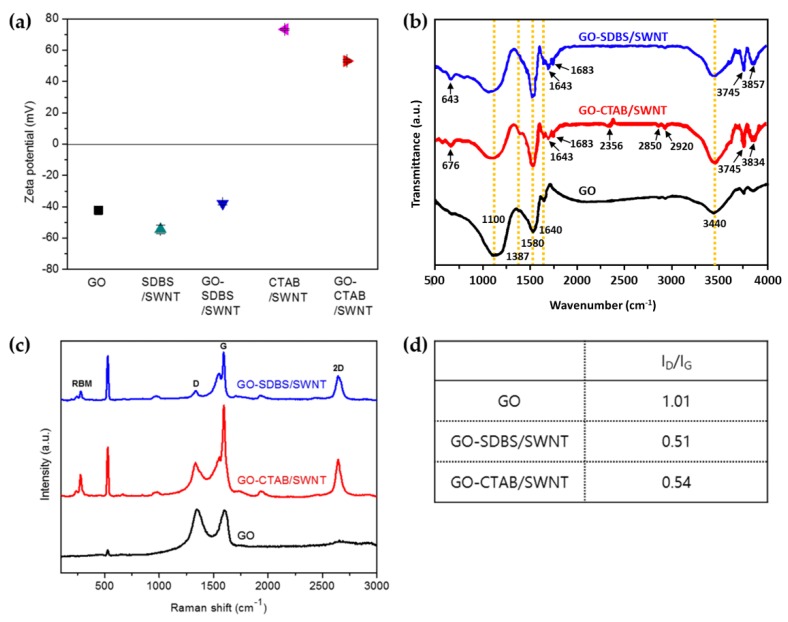
The results of (**a**) Zeta-potential, (**b**) FT-IR spectra, (**c**) Raman spectra, and (**d**) I_D_/I_G_ values of GO, GO-SDBS/SWNT, and GO-CTAB/SWNT.

Figure 5a shows the zeta-potential of the GO, SDBS/SWNT, GO-SDBS/SWNT, CTAB/SWNT, and GO-CTAB/SWNT to identify a suitable dispersion and connection between CNT with each surfactant and GO. Primarily, the zeta-potential is a vital indicator of the stability of colloidal dispersions. According to the ASTM D4187-8 for the stability of colloidal suspensions, the zeta-potential in the region more than 30 mV (of either positive or negative) is regarded as moderately stable [26]. The magnitude of the zeta-potential of all samples is more than 30 mV, so they are stable colloids.

Additionally, the surfaces of GO sheets are highly negatively charged, apparently as a result of the existence of the carboxylic and hydroxyl groups on these sheets. Although CNT is a neutral, SDBS/SWNT and CTAB/SWNT are negatively and positively charged, respectively. For this reason, there could be a tight connection between each surfactant and CNT. In cases of GO-SDBS/SWNT and GO-CTAB/SWNT, they are also negatively and positively charged, respectively. However, the magnitude of these hybrid samples decreased. From this, they have a good connection between CNT with each surfactant and GO.

As shown in Figure 5b, FT-IR was also used to confirm the proper connections. For the GO, there is a broad absorption band with a peak at 3300 cm^−1^ due to the stretching vibration of O–H, while the peak at 1717 cm^−1^ is attributed to C=O bond stretching. The deformation band at 1387 cm^−1^ indicated tertiary C–OH, while the peaks at 1042 cm^−1^ and 1220 cm^−1^ belong to C–O of alkoxy and epoxy bond, respectively. The other bands at 2975 cm^−1^ and 1617 cm^−1^ indicate the C–H stretching of aliphatic groups and C=C bonds.

On the other hand, for both of GO-SDBS/SWNT and GO-CTAB/SWNT, the characteristic peaks at 3720 cm^−1^, 2870–2950 cm^−1^, and 1470–1520 cm^−1^ correspond to slightly shifted O–H, aromatic with =C–H and ring C=C stretching vibration, and with –CH_3_ and –CH_2_ stretching vibration, respectively. These peaks are due to the attachment of surfactant molecules. Specifically, it was determined that CNT exist in GO-SDBS/SWNT and GO-CTAB/SWNT hybrid foams when compared with FT-IR spectra of CNT [27].

In addition to SEM images shown in Figure 4, the porous structure of the resulting GO-SWNT hybrid foams was revealed by Raman spectra which are shown in Figure 5c. There are Raman spectra results of GO-SDBS/SWNT, GO-CTAB/SWNT hybrid foam and GO foam.

In case of GO foam, it had two prominent peaks at ~1350 cm^−1^ and ~1580 cm^−1^ corresponding with D and G bands due to C–C stretching of the graphitic lattice. In comparison, the Raman spectra of GO-SWNT hybrid foams indicated the distinctive D, G, 2D, and radial breathing mode (RBM) bands of CNTs. In addition to the presence of the D band, the G band of the GO-SWNT hybrid split into two bands, called G^+^ and G^−^ due to the effect of curvature on the nanotube G peak [28]. The new 2D band at 2700 cm^−1^ can be used to determine the amount of disorder. It implies that the disorder caused by oxidation and reduction of GO is partially recovered by adding SWNTs. In addition, the RBM is representative of SWNTs due to an isotropic radial vibration in a range from 120 cm^−1^ to 350 cm^−1^.

Besides the 2D band, the integrated intensity ratio between the D and G bands (I_D_/I_G_) is denoted as a structural disorder; an increase in the I_D_/I_G_ ratio is attributed to an increase in the disorder [29]. Figure 5d shows the I_D_/I_G_ ratio of GO, GO-SDBS/SWNT, and GO-CTAB/SWNT. The I_D_/I_G_ ratio of two kinds of the GO-CNT hybrid foams is much smaller than that of GO foams. It also means that the crystallinity is recovered.

**Figure 6:**
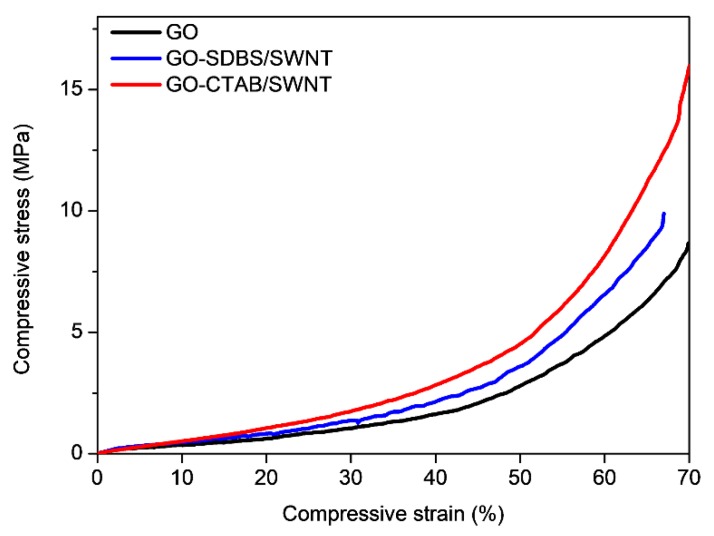
Stress–strain curves of GO, GO-SDBS/SWNT, and GO-CTAB/SWNT under compression.

To investigate the reinforcing effect of CNT depending on two different ionic surfactants, monotonic compression tests were conducted according to ASTM D1621-10. GO-CTAB/SWNT, GO-SDBS/SWNT, and GO foam are ranked in order of largest compressive modulus and strength to the smallest. In detail, the compressive modulus of GO-CTAB/SWNT hybrid foam was 157% higher than GO foam. Furthermore, the strength at 10% strain of GO-CTAB/SWNT hybrid foam was 54.3% higher than GO foam. CNT prevents the graphene walls from collapsing under compressive stress, so CNT reinforced hybrid foams have higher compressive modulus and strength than GO. Moreover, GO-CTAB/SWNT has higher Young’s modulus (E) and strength than GO-SDBS/SWNT because the interaction between GO and CTAB/SWNT is ionic bonding and π–π interaction, but GO and SDBS/SWNT is only π–π interaction. Therefore, CTAB/SWNT is more effective reinforcement for GO foams than SDBS/SWNT (Figure 6, Table 1).

## 4. Conclusions

Although graphene has superior properties, such as chemical and mechanical properties, it has not been commercialized or translated into practical outcomes, yet. Recently, 3D graphene architectures have been attracting significant interests as a key solution to solve problems, such as low productivity, high cost, difficulty in controlling high-quality materials, defects and dispersion problem. However, recent researches on 3D graphene architectures struggle with strength. To solve this obstacle, we synthesized Graphene-CNT hybrid foams by introducing CNT as a reinforcement. In addition, when CNT dispersions were processed, a strategy of interaction was applied by using anionic and cationic surfactants.

This study confirmed that GO-CNT hybrid foams had good connections between CNT with each surfactant and GO. The charge effect of surfactants, covered CNT, was experimentally investigated. Furthermore, by applying CNT as a reinforcement, the compressive modulus and strength at 10% strain dramatically increased. Therefore, it can be concluded that the CNT with different ionic surfactants as reinforcement can act as a good reinforcement. Moreover, it was experimentally investigated that the CTAB/SWNT is a more effective reinforcement for GO foams because the formation of ionic bonding between GO and CTAB/SWNT is stronger than the π–π interaction induced from GO and SDBS/SWNT.

## Figures and Tables

**Table 1:** Comparison of Young’s modulus and strength of GO, GO-SDBS/SWNT, and GO-CTAB/SWNT.

Property	GO	GO-SDBS/SWNT	GO-CTAB/SWNT	% Increased
E (MPa)	35	65	90	(157% increase)
Strength at 10% strain (MPa)	0.35	0.52	0.54	(54.3% increase)
